# Genetic Variation and Spatial Genetic Structure of *Eleocharis ussuriensis* Zinserl. in South Korea: Implications for Ecological Monitoring and Resource Management

**DOI:** 10.3390/genes17050513

**Published:** 2026-04-26

**Authors:** Eun-Hye Kim, Kang-Rae Kim, Mi-Hwa Lee, Jaeduk Goh, Jeong-Nam Yu

**Affiliations:** 1 Nakdonggang National Institute of Biological Resources, Sangju 37242, Republic of Korea; karpo83@nnibr.re.kr (E.-H.K.); blume96@nnibr.re.kr (M.-H.L.); jdgoh@nnibr.re.kr (J.G.); 2 Southeast Sea Fisheries Research Institute, National Institute of Fisheries Science, Namhae 52440, Republic of Korea; kimkangrae9586@gmail.com

**Keywords:** *Eleocharis ussuriensis*, microsatellite (SSR), genetic diversity, genetic differentiation, population structure

## Abstract

**Background/Objectives**: *Eleocharis ussuriensis* Zinserl. is a perennial riparian sedge widely distributed in Northeast Asia and a dominant component of freshwater vegetation in South Korea. However, the intraspecific genetic structure of this species across contrasting hydrological habitats remains insufficiently understood. This study aimed to develop novel SSR markers from whole-genome data and investigate genetic variation and population structure among *E. ussuriensis* populations in South Korea. **Methods**: Twenty-one novel simple sequence repeat (SSR) markers were developed from whole-genome sequence data and applied to analyze genetic variation in 120 individuals from 6 populations. Genetic diversity, differentiation, and gene flow were estimated using allele-frequency-based metrics, and population genetic structure was further evaluated using spatial information derived from geographic coordinates. **Results**: A total of 201 alleles were detected, with a mean polymorphism information content (PIC) of 0.759, indicating high marker informativeness. Mean genetic diversity across populations showed observed heterozygosity (Ho = 0.360) and expected heterozygosity (He = 0.281), while multilocus genotype ratios (G/N) ranged from 0.30 to 1.00 among populations. Genetic differentiation was substantial (F_ST_ = 0.373–0.669; Jost’s D = 0.540–0.997). Mantel tests revealed that genetic differentiation was significantly correlated with geographic distance (*r* = 0.67, *p* < 0.001). Both allele-frequency-based and spatially explicit approaches suggested genetic structuring among populations. **Conclusions**: The results suggest spatial tendencies in genetic structure among populations, reflecting patterns of allele distribution across regions. These findings provide baseline information on genetic variation in *E. ussuriensis* and may contribute to a better understanding of its ecological dynamics.

## 1. Introduction

River ecosystems are increasingly recognized for their critical ecological functions under rapid environmental change driven by climate change [1,2]. From a landscape ecological perspective, river systems function as dendritic networks that connect individuals and genes through water-mediated dispersal pathways [3]. Hydrological disturbances, such as flooding, may contribute to heterogeneous genetic structures in riparian vegetation by influencing dispersal, establishment, and survival processes [4,5]. Because riparian plants are closely linked to river dynamics, they are directly affected by environmental variables, including water quality, water-level fluctuations, and flow velocity [6]. In frequently disturbed or environmentally unstable habitats, opportunities for sexual reproduction may be reduced, and repeated clonal expansion of a limited number of genotypes can lower population-level genetic diversity [7]. Riparian and aquatic plants often possess functional traits that enable persistence under such fluctuating conditions, notably clonal growth strategies that enhance survival in saturated and disturbance-prone environments [8].

Although numerous studies have examined the ecological functions and species diversity of riverine systems [9], comparatively less attention has been given to intraspecific genetic variation in widespread and non-threatened plant species, as population genetic research has often focused on endangered or rare taxa. Population genetic approaches provide effective tools for evaluating these intraspecific processes by quantifying genetic diversity, population structure, and differentiation. Such information is essential for understanding the effects of habitat fragmentation, defining conservation units, and establishing genetic baselines for resource management. Notably, riparian plant populations distributed across contrasting hydrological environments may exhibit distinct patterns of genetic differentiation associated with differences in hydrological connectivity [10].

*Eleocharis ussuriensis* Zinserl. is a perennial riparian species distributed across wetlands in Northeast Asia, including Korea, China, and Japan [11,12]. The species belongs to the family Cyperaceae and is wind-pollinated, while congeners are known to exhibit mixed reproductive strategies, including both selfing and outcrossing. In South Korea, *E. ussuriensis* typically occurs along calm water margins or reservoir edges. Through rhizomatous growth and the formation of dense clonal stands, this species contributes to sediment stabilization and the maintenance of riparian ecosystem structure (Figure S1) [13]. Such life-history traits imply the potential for localized clonal expansion alongside broader dispersal via pollen or propagule movement [14]. Beyond its ecological role, extracts of *E. ussuriensis* have been patented for cosmetic applications in South Korea due to reported skin-whitening properties and are registered in the International Cosmetic Ingredient Dictionary [15]. Despite traits that may suggest considerable dispersal potential, it remains unclear whether effective gene flow is maintained across hydrological landscapes and whether this potential dispersal translates into stable genetic connectivity among populations inhabiting contrasting hydrological environments.

The family Cyperaceae is widely recognized as taxonomically challenging due to morphological similarity and limited diagnostic characteristics [16]. Within this family, the genus *Eleocharis* is particularly difficult to classify, as its morphological simplicity and high variability of diagnostic traits result in substantial variation both within and among species [17,18,19].

To address these taxonomic limitations and ensure reliable genetic monitoring, robust molecular tools are required. SSR-based analyses have been conducted in related species such as *E. dulcis* [20,21], and some studies suggest that microsatellite markers may be transferable across closely related species [22]. However, their applicability remains limited and context-dependent. Previous studies have shown that even within the same genus, the success rate of cross-amplification is often moderate (approximately 60%), depending on genetic similarity among taxa [23]. Although cross-species amplification may be achieved, substantial interspecific variation in polymorphism levels often limits the reliability and overall informativeness of these markers [22]. Despite these limitations, no species-specific microsatellite markers have been developed for *E. ussuriensis* to date. Therefore, the development of species-specific markers is essential for obtaining accurate and robust genetic information.

Accordingly, this study aimed to develop novel species-specific microsatellite (SSR) markers for *E. ussuriensis* based on whole-genome sequence data in order to improve marker stability and overcome the limitations of markers derived from restricted genomic regions. Using these SSR markers, we assessed genetic diversity, population differentiation, and spatial genetic structure to establish a genetic framework for population discrimination and provide baseline genetic information for the future management and utilization of this species.

## 2. Materials and Methods

### 2.1. Collection of Populations

**Figure 1:**
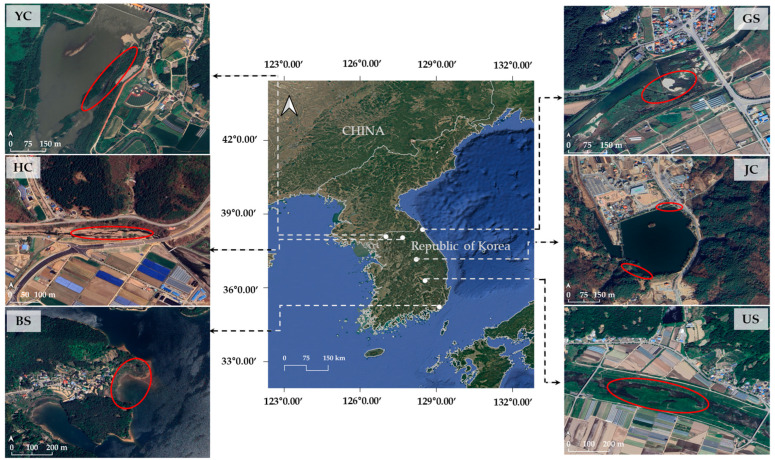
Geographic distribution of six populations of *Eleocharis ussuriensis* in South Korea. The central map shows the overall locations of the sampling sites (white dots). Surrounding panels present detailed satellite images of each population (YC, HC, GS, JC, US, and BS), with red ellipses indicating the sampling areas. Scale bars are provided in each subfigure. Scale 1: 2000 to 3000.

In May 2023, a total of 120 individuals of *E. ussuriensis* were collected from six riparian populations across South Korea (20 individuals per population; Figure 1). To evaluate both the discriminatory power of the markers and patterns of clonal diversity, sampling sites were strategically selected from independent drainage systems and contrasting habitats. Specifically, four populations were located in lotic (riverine) environments—YC (Yeoncheon; 38°5′55.42″ N, 127°0′57.97″ E), HC (Hwacheon; 38°3′22.60″ N, 127°40′34.85″ E), GS (Goseong; 38°23′13.4″ N, 128°27′42.1″ E), and US (Uiseong; 36°18′44.1″ N, 128°33′41.1″ E)—whereas two populations represented lentic (reservoir) habitats—JC (Jecheon; 37°10′31.4″ N, 128°12′42.4″ E) and BS (Busan; 35°14′51.04″ N, 129°7′2.53″ E) (Table S1). To avoid collecting genetically related individuals arising from clonal growth or restricted pollen dispersal, a minimum distance of 5 m was maintained between sampled individuals within each population. All voucher specimens are housed at the Nakdonggang National Institute of Biological Resources, South Korea (Voucher Nos. NNIBRVP127067–127186).

### 2.2. Microsatellite (SSR) Marker Development

Field-collected leaves were silica-dried and stored at −80 °C prior to genomic DNA extraction using the DNeasy Plant Mini Kit (QIAGEN, Hilden, Germany). SSR loci were identified from the assembled genome (NCBI BioSample: SAMN55324966) using MISA, with minimum repeat thresholds of 4–10 units. Primer3 generated 100 initial primer pairs (amplicon: 100–300 bp, length: 20–24 bp, GC: 40–60%, Tm: 60 °C), whose binding specificities were validated via SnapGenev7.1.0 (GSL Biotech, Chicago, IL, USA). Primer screening was performed on six individuals per population, retaining only loci with clear and reproducible amplification. The validated microsatellite markers were deposited in GenBank (accession numbers PX920838–PX920858; Table S2).

PCR was performed using a Mastercycler Pro Gene Amplifier (Eppendorf, Hamburg, Germany) and H-Star Taq DNA Polymerase (Biofact, Daejeon, Republic of Korea). Following the M13-tailed method [24], 20 μL reactions contained 20–50 ng genomic DNA, 0.4 μM locus-specific forward primer, 0.08 μM M13-tailed reverse primer, and 0.4 μM fluorescently labeled M13 primer (6-FAM, VIC, NED, or PET). Cycling conditions were: 94 °C for 5 min; 30 cycles of 94 °C for 30 s, 60 °C for 45 s, and 72 °C for 45 s; 12 cycles of 94 °C for 30 s, 53 °C for 45 s, and 72 °C for 45 s; and a final extension at 72 °C for 10 min. Amplicons were mixed with GeneScan™ (Thermo Fisher Scientific, Waltham, MA, USA) 500 ROX and Hi-Di™ (Thermo Fisher Scientific, Waltham, MA, USA) formamide, denatured at 95 °C for 2 min, cooled to 4 °C, and analyzed using an ABI 3730xl DNA Analyzer (Thermo Fisher Scientific, Waltham, MA, USA). Allele scoring was performed using GeneMarker^®^ v2.6.7 (SoftGenetics, State College, PA, USA).

### 2.3. Genetic Diversity and Bottleneck Analysis

To ensure data quality, MICROCHECKER v2.2.3 [25] was used to detect potential genotyping errors, including stuttering, large-allele dropout, and null alleles. This evaluation was based on deviations from expected heterozygote frequencies and repeat motif patterns via 1000 Monte Carlo iterations at a 95% confidence interval (CI). Standard genetic diversity indices, including the percentage of polymorphic loci (P0.95), mean (A) and effective (Ae) number of alleles, and observed (Ho) and expected (He) heterozygosity, were calculated in PopGene v1.32 [26]. Additionally, polymorphism information content (PIC) was determined using Cervus v3.0.7 [27]. To examine the distribution of shared and population-specific alleles across the 21 microsatellite loci, allele frequency data from each sampling site were visualized using the R package UpSetR v1.4.0 [28].

Population differentiation (*F*-statistics) was estimated using the R package hierfstat v0.5-11 [29], with statistical significance tested through permutation procedures in FSTAT v2.9.4 [30]. To identify recent bottleneck signals, BOTTLENECK v1.2.02 [31] was employed. Statistical significance was evaluated via a one-tailed Wilcoxon signed-rank test (10,000 iterations) under three mutation models: the infinite alleles model (IAM), the stepwise mutation model (SMM), and a two-phase model (TPM). Notably, the TPM (configured with 70% SMM, 30% IAM, and variance = 12) was applied as the primary model.

### 2.4. Genetic Differentiation and Gene Flow

To quantify genetic differentiation among populations, Wright’s *F*-statistics (F_IS_ and F_ST_; [32]) were calculated using FSTAT v2.9.4 [30], with statistical significance evaluated via 180,000 permutations. Population structure was further assessed through analysis of molecular variance (AMOVA) in GenAlEx v6.503 [33]. Additionally, Jost’s *D* (Dest) [34] was computed, with 95% confidence intervals derived from 1000 permutations and bootstrap resampling. Genetic relationships among populations were visualized through principal coordinate analysis (PCoA). The relationship between genetic differentiation and geographic distance was examined using Mantel tests [35] implemented in IBD software v1.52 [36]. Directional gene flow was estimated via the DivMigrate online tool [37], part of the *diveRsity* package [38], based on 1000 bootstrap replicates.

### 2.5. Spatial and Genetic Structure

Population genetic structure was analyzed using STRUCTURE v2.3.4 [39] under the admixture model with correlated allele frequencies [40,41]. To determine the optimal number of clusters (K), values ranging from 1 to 10 were tested. For each K, 10 independent runs were performed, each comprising 100,000 burn-in iterations followed by 1,000,000 Markov chain Monte Carlo (MCMC) iterations; the optimal K value was determined using the StructureSelector web tool [42].

Spatial genetic structure was further examined using TESS3r [43], a spatial clustering approach based on sparse non-negative matrix factorization that incorporates individual geographic coordinates. Analyses were conducted with K values ranging from 1 to 10 (10 replicates per K), and the optimal K was identified using the cross-entropy criterion. Spatial patterns of genetic structure were then visualized based on the dominant membership coefficients (Q values) assigned to each individual across both STRUCTURE and TESS3r analyses.

## 3. Results

### 3.1. Variation in Microsatellite Loci

A total of 201 alleles were detected across 21 microsatellite loci. The number of alleles per locus ranged from 4 to 18, with a mean of 9.6. Polymorphism information content (PIC) values varied among markers, ranging from 0.465 (EU57) to 0.882 (EU82), with an average of 0.759, indicating a generally high level of polymorphism (Table 1). Observed heterozygosity (Ho) ranged from 0.117 (EU05 and EU86) to 0.667 (EU100), with a mean of 0.362, whereas expected heterozygosity (He) ranged from 0.551 (EU57 and EU86) to 0.895 (EU82), with a mean of 0.790.

Locus-specific FIS values varied from −0.935 (EU100) to 0.683 (EU05), and heterozygote excess was observed at several loci. Locus-specific FST values ranged from 0.339 (EU04) to 0.887 (EU86), with a mean of 0.639, indicating strong genetic differentiation among populations.

### 3.2. Genetic Diversity and Bottleneck

**Figure 2:**
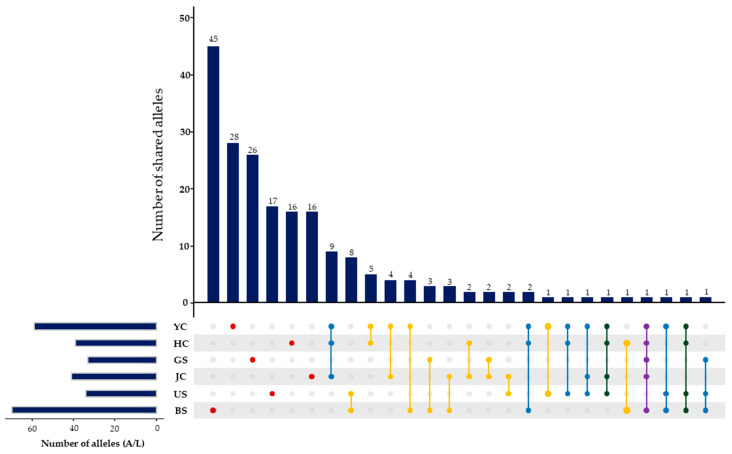
Allele sharing patterns and unique alleles among 6 populations of *E. ussuriensis* (YC, HC, GS, JC, US, and BS). Distinct colors indicate different allele-sharing patterns across populations.

We analyzed genetic diversity across the 6 populations using the 21 microsatellite loci, detecting 201 alleles (Table 2). The observed numbers of alleles per population ranged from 33 (GS) to 70 (BS). Analysis of allele sharing among the populations revealed 148 alleles were population-specific, with each population harboring 16 (HC and JC) and 45 (BS) alleles. In addition, we identified 53 widespread alleles shared by at least two populations (Figure 2).

The proportion of polymorphic loci (P_0_._95_) values ranged from 33.3 (GS) to 76.2 (YC and BS) (Table 2). The effective number of alleles per locus (Ae/L) varied from 1.4 (GS and JC) to 2.2 (BS). Observed heterozygosity (Ho) ranged from 0.155 (GS) to 0.645 (HC), whereas expected heterozygosity (He) ranged from 0.163 (GS) to 0.432 (BS). The proportion of multilocus genotypes (G/N) observed in each population ranged from 0.30 (US) to 1.00 (BS).

Across all populations, the mean observed heterozygosity (Ho) was 0.362, the mean expected heterozygosity (He) was 0.284, and the mean effective number of alleles per locus (Ae/L) was 1.7. The mean multilocus genotype ratio (G/N) was 0.80.

The inbreeding coefficient (F_IS_) indicated excess heterozygosity in most populations, with values ranging from −0.840 (HC) to 0.076 (GS), while a slightly positive F_IS_ value was observed in GS (0.076) (Table 3). The HC population exhibited strong bottleneck signals under all three mutation models (*p* < 0.001). In contrast, significant heterozygosity excess under the IAM and TPM was detected in the GS, US, and BS populations, whereas no significant bottleneck signals were observed in the YC and JC populations. Overall, results under the SMM were weaker and less consistent.

### 3.3. Genetic Differentiation and Gene Flow

**Figure 3:**
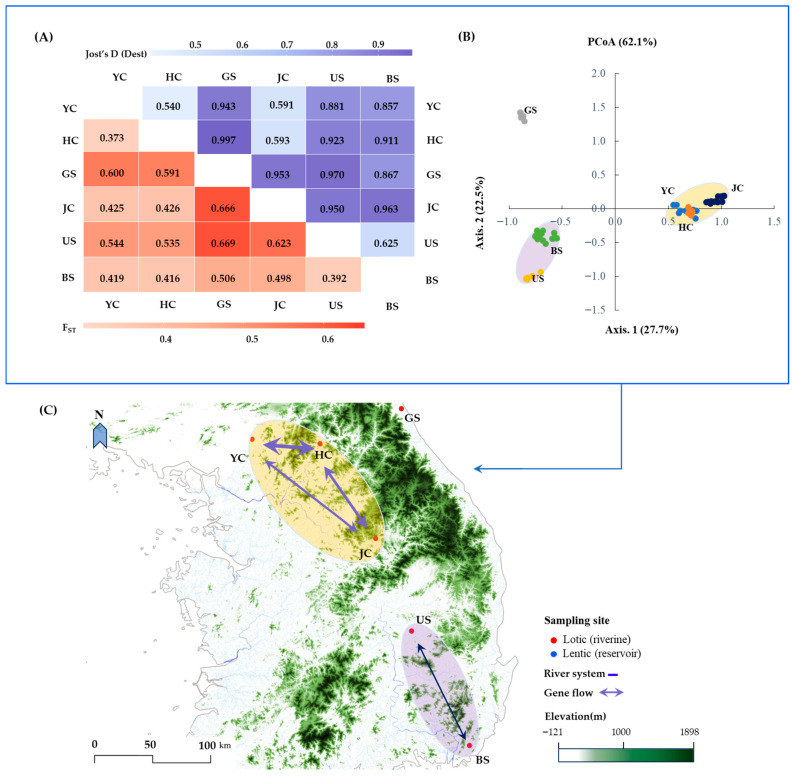
Genetic differentiation and spatial structure among 6 populations (YC, HC, GS, JC, US, and BS) of *E. ussuriensis*. (**A**) Pairwise genetic differentiation based on F_ST_ (lower triangle) and Jost’s D (upper triangle). (**B**) Principal coordinate analysis based on microsatellite data. Ellipses represent groups of populations that show concordant patterns in both PCoA clustering and directional gene flow analysis. (**C**) Geographic distribution of sampling sites with inferred genetic connectivity patterns within river systems.

Analysis of molecular variance revealed that 62% of the total genetic variation was partitioned among populations, whereas the remaining variation occurred within individuals. This result indicates substantial genetic differentiation among the 6 populations. Moreover, pairwise F_ST_ values ranged from 0.373 (YC and HC) to 0.669 (US and GS), indicating moderate to high genetic differentiation among the populations (Figure 3A). Consistent with the F_ST_ results, pairwise genetic differentiation based on Jost’s D (Dest) revealed high levels of differentiation, ranging from 0.540 (JC and HC) to 0.997 (GS and HC). The Mantel test revealed a statistically significant positive correlation between genetic and geographic distances (*r* = 0.67, *p* < 0.001; Table S3).

Principal coordinate analysis (PCoA) reflected the pattern of genetic differentiation among populations, with the first two axes explaining 50.2% of the total genetic variation (Axis 1: 27.7%; Axis 2: 22.5%), and the cumulative variation reaching 62.1% across the first three axes (Axis 3: 11.9%). Along the first axis, YC, HC, and JC were grouped on the right side, whereas US and BS were positioned on the left side. The GS population was clearly separated from the other populations, forming a distinct cluster in the upper-left region of the plot (Figure 3B). This pattern was further supported by the directional migration analysis, which revealed clustered gene flow among populations. Bidirectional migration among YC, HC, and JC showed high migration coefficients, with the highest value observed between YC and HC (migration coefficient = 1.00). US and BS also exhibited reciprocal migration (0.39–0.45), whereas GS consistently showed lower migration values than the other populations (Figure 3C and Table S3).

### 3.4. Spatial and Genetic Structure

**Figure 4:**
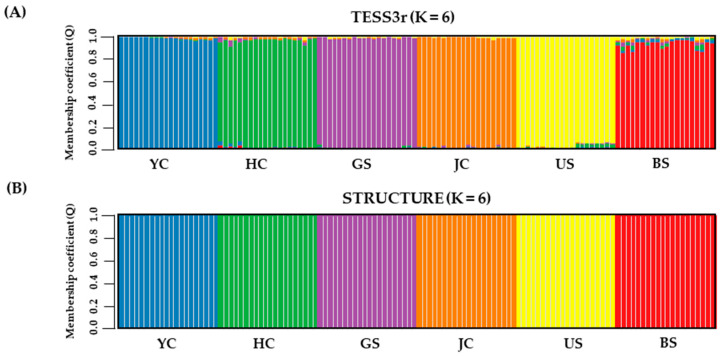
Genetic structure of six *E. ussuriensis* populations (K = 6) inferred using (**A**) TESS3r and (**B**) STRUCTURE. Vertical bars represent individuals, and colors indicate their membership coefficients (Q-values) for genetic clusters. Populations are grouped sequentially: YC, HC, GS, JC, US, and BS. Colors correspond to the six genetic clusters, and mixed colors within a single bar indicate admixture.

Genetic structure analysis of the 6 populations indicated an optimal number of clusters (K = 6), as inferred from the allele-frequency-based clustering model (STRUCTURE). The spatially explicit approach (TESS3r) also supported an optimal number of clusters of 6 (Figure 4). At this clustering level, individuals were largely assigned to clusters corresponding to their sampled populations, indicating well-defined population-level genetic structuring.

## 4. Discussion

### 4.1. Variation in Microsatellite Loci

Several microsatellite loci developed in this study showed high levels of polymorphism (PIC = 0.759); however, some loci exhibited extreme F_IS_ values (e.g., EU05 and EU100) and significant deviations from the Hardy–Weinberg equilibrium, which were primarily associated with regionally fixed or nearly fixed alleles (Table 1 and Figure 2). These patterns may be partly attributable to reproductive characteristics of Cyperaceae, including limited pollen and seed dispersal and clonal reproduction, as well as to region-specific population differentiation [44,45].

Distinct allele patterns observed in *E. ussuriensis* may also reflect broader genomic characteristics of the genus. Species of *Eleocharis* are known to possess holocentric chromosomes and dynamic repetitive DNA fractions, features that have been associated with the rapid turnover of repetitive sequences [46]. Although this study did not directly assess repeat landscape dynamics, such genomic properties may contribute to microsatellite variability [47], in addition to demographic processes such as genetic drift, clonal reproduction, and restricted gene flow.

Importantly, while recent advances in genomic technologies—such as whole-genome resequencing and reduced-representation sequencing approaches—have greatly expanded the resolution of population genetic analyses, the development of stable and transferable marker systems remains essential [48]. High-throughput genomic methods can generate extensive datasets; however, the comparability of results across temporal scales and independent studies often depends on the use of standardized and reproducible markers [49]. In this context, microsatellite markers continue to provide practical advantages, including cost-efficiency, methodological consistency, and suitability for long-term monitoring frameworks [50]. Therefore, the SSR markers developed in this study are expected to serve as robust genetic tools for assessing population-level variation and for enabling future comparative analyses across space and time in *E. ussuriensis*.

### 4.2. Genetic Diversity and Bottleneck

The mean observed and expected heterozygosity values (Ho = 0.362 and He = 0.284) of *E. ussuriensis* in this study were lower than those reported for the congeneric species *E. parvula* (Ho = 0.521, He = 0.404) [51]. When expanding the comparison to the broader Cyperaceae family, the genetic diversity of *E. ussuriensis* was found to be comparable to or slightly lower than that of species such as *Carex scabrifolia* (Ho = 0.350, He = 0.419) [52] and *Carex kobomugi* (Ho = 0.648, He = 0.451) [53]. Previous studies have also reported relatively high levels of genetic diversity in other *Eleocharis* species, such as *E. acuta* [54] and *E. tuberosa* [55].

However, direct comparisons of genetic diversity among studies are inherently limited due to differences in analytical approaches and molecular marker systems (e.g., the use of ISSR markers in *E. tuberosa* [55]). Diversity indices derived from different marker types are not directly comparable; therefore, only general trends should be interpreted with caution. Furthermore, although the family Cyperaceae comprises a large number of species, population genetic studies based on microsatellite markers remain relatively limited [22], and comparative information on genetic diversity across species is still scarce.

Beyond methodological differences, the observed genetic patterns may also reflect underlying environmental heterogeneity, including variation in habitat extent, population size, and disturbance regimes. Many aquatic plants reproduce predominantly through clonal growth and often exhibit high clonal diversity [56]. In particular, clonal plant populations in riverine systems are often influenced by hydrological disturbance and directional water flow, which can restrict sexual recruitment while promoting the persistence and spread of dominant genotypes, a pattern commonly observed in clonal riparian species such as *Phragmites australis* [57]. Such diversity may arise from occasional sexual recruitment or be maintained through long-term vegetative persistence, particularly in relatively stable environments [58]. These patterns are commonly associated with habitats characterized by low hydrological disturbance, such as ponds and lakes [59,60].

Taken together, these results highlight the need to accumulate genetic information not only within the genus *Eleocharis* but also across the Cyperaceae family, in order to support the conservation and sustainable use of plant genetic resources.

Among the four lotic populations analyzed in this study, HC, GS, and US exhibited lower levels of genetic diversity compared to YC, with multilocus genotype (MLG) ratios ranging from 0.30 to 0.40, below the overall population means. In addition, bottleneck signals were detected under all three mutation models, suggesting that these populations may have experienced recent demographic fluctuations or disturbance events. These populations are located in riverine environments in South Korea, where seasonal flooding frequently occurs during the summer monsoon period [61,62]. Such hydrological disturbances, together with anthropogenic activities such as riverbank modification for flood control, may influence population dynamics and could contribute to clonal propagation [59,63,64].

This contrast was evident in the distinct genetic characteristics of the two tributary populations. YC exhibited a high proportion of multilocus genotypes and balanced levels of heterozygosity, suggesting sustained recruitment of genetically diverse individuals. In contrast, HC showed pronounced heterozygote excess and significant bottleneck signals (Table 3), consistent with recent demographic instability or recruitment events dominated by a limited number of genotypes. These results suggest that local population history and establishment success play a key role in shaping genetic diversity in tributaries.

The JC population occurs in an older reservoir that has been maintained as a recreational and tourism site under local government management, and may therefore be relatively buffered from natural environmental fluctuations. Consistent with this, no clear bottleneck signal was detected in this population. However, its relatively low genetic diversity compared to the overall population means, along with a low G/N value, may reflect limited population expansion, potentially associated with management practices in the surrounding reservoir environment.

In contrast, the BS population exhibited relatively high genetic diversity. Despite bottleneck signals being detected under some mutation models, all sampled individuals possessed unique genotypes. This population occurs within a protected drinking-water reservoir, where intensive management is implemented, and anthropogenic disturbance is therefore relatively limited. Furthermore, the reservoir was formed by the convergence of two hydrological systems. Together, such unique hydrological features and stable habitat conditions may contribute to the maintenance and accumulation of genetic variation within the population [65,66]. Additionally, natural hybridisation among *Eleocharis* species has been reported [67], which may also be associated with the observed genetic diversity.

These results suggest that genetic variation in *E. ussuriensis* populations may be strongly influenced by habitat conditions, similar to patterns observed in other aquatic plant species. Accordingly, long-term monitoring is needed to better understand the factors contributing to population-level genetic characteristics.

### 4.3. Genetic Differentiation and Gene Flow

Hydrological conditions and topographic features are well known to influence patterns of gene flow in riverine systems [68]. In addition, species-specific life-history traits, including reproductive mode and dispersal capacity, may further reinforce barriers to gene exchange, resulting in fine-scale and heterogeneous genetic structuring [69]. In riparian habitats, such interacting environmental and biological factors are generally expected to produce moderate levels of spatial genetic differentiation.

AMOVA results indicated that 62% of the total genetic variation was partitioned among populations, and pairwise estimates further demonstrated substantial differentiation (F_ST_ = 0.373–0.669; Jost’s D = 0.540–0.997; Figure 3A). Although a significant isolation-by-distance (IBD; r = 0.67, *p* < 0.001) pattern was detected, suggesting that geographic distance contributes to genetic divergence, the magnitude of differentiation exceeds what would be expected from distance alone [70]. The generally high differentiation values indicate limited allele sharing among populations. This suggests that genetic divergence is not solely driven by differences in allele frequencies but also by the presence of population-specific alleles, implying restricted effective gene flow and localized genetic turnover [71].

To further evaluate whether geographic distance alone explains the observed differentiation, we examined patterns of directional migration among populations. Directional migration analysis revealed limited and asymmetric gene flow across the study area. Strong reciprocal gene flow was detected among the YC, HC, and JC populations, indicating ongoing or recent genetic connectivity within this regional cluster (Figure 3C). In particular, gene flow between the YC and HC populations was the strongest (relative migration rate = 1.00; Table S3), which may reflect relatively high genetic connectivity despite their occurrence in different tributaries. However, as noted earlier, such high propagule exchange does not necessarily lead to genetic homogenization; rather, it may reflect local environmental filtering or recent bottlenecks that limit successful establishment in specific habitats, such as HC.

Interestingly, despite lacking direct hydrological connectivity with other populations, populations in isolated environments can still exhibit genetic patterns shaped by complex dispersal mechanisms. For instance, the JC population, located in an isolated reservoir, still exhibited a genetic pattern consistent with geographic distance. Although information on *E. ussuriensis* remains limited, studies on related *Eleocharis* species (e.g., *E. acuta* and *E. sphacelata*) have demonstrated high dispersal potential associated with seed buoyancy and dormancy under unfavorable environmental conditions [72]. Furthermore, animal-mediated dispersal has been widely reported in aquatic plants [73], suggesting that biotic vectors such as waterbirds may facilitate overland seed movement between unconnected water bodies. Such mechanisms may explain the moderate but directional migration observed between the US and BS populations, suggesting localized connectivity despite their geographic separation and limited hydrological linkage. However, the exact role of these vectors was not directly assessed in the present study and requires further investigation.

In contrast, gene flow involving the GS population was consistently low, supporting its relatively isolated genetic position. This isolation may be associated with large-scale topographical features that limit hydrological connectivity and potential dispersal pathways among systems (Figure 3C).

Together, these findings reinforce the fact that gene flow in *E. ussuriensis* is spatially structured and heterogeneous. Genetic connectivity varies markedly among specific population pairs due to the interplay of landscape features, local environmental conditions, and potential dispersal vectors, rather than following a simple distance-dependent or uniform pattern across the landscape [74].

### 4.4. Spatial and Genetic Structure

The substantial genetic differentiation and restricted gene flow identified among populations were consistently reflected in both allele-frequency-based and spatially explicit clustering analyses (Figure 4). Each population formed a distinct genetic cluster, indicating strong population-level structuring across the study area. The presence of private alleles and distinct genetic compositions across all six populations, together with the allele-sharing patterns revealed by the UpSet plot (Figure 2), further supports the interpretation that each population represents a genetically distinct unit with limited gene flow among populations.

The patchy distribution and clonal growth form of *E. ussuriensis* likely contribute to this pattern [75,76], as clonal propagation and localized recruitment in aquatic and riparian plants can limit effective dispersal and enhance genetic differentiation among populations [56,59,65]. In clonal riparian species, limited effective dispersal and localized recruitment can restrict gene flow and promote genetic differentiation among sites, even within seemingly connected riverine landscapes [77,78]. This interpretation is consistent with our AMOVA results, which demonstrated that genetic variation is largely partitioned among populations (Φ_ST_ = 0.62), rather than within them.

The significant isolation-by-distance pattern further supports the view that geographic distance and localized processes jointly shape the spatial genetic structure in this species [79]. Even in the absence of direct hydrological connectivity, limited gene flow may still occur at local spatial scales, likely facilitated by alternative dispersal vectors or historical connectivity.

### 4.5. Resource Management

The genetic differentiation and spatial structuring of *E. ussuriensis* observed in this study suggest that each regional population may function as a partially independent genetic unit rather than a homogeneous group. The limited sharing of alleles among populations may reflect both reproductive characteristics and environmental influences, including habitat conditions and hydrological connectivity [80].

Furthermore, because it remains unclear whether the observed genetic variations directly contribute to physiological or morphological traits, long-term monitoring will be valuable to minimize potential errors in morphological classification and further evaluate the adaptive potential of this species as a biological resource. From a resource management perspective, the presence of private alleles within each population (Figure 1) and the high level of genetic differentiation (Figure 3A) observed in this study highlight the importance of maintaining the genetic distinctiveness of local populations. Accordingly, the indiscriminate translocation or artificial mixing of plant materials among different regions should be approached with caution, as it may not only reduce genetic differentiation among populations but also weaken the distinct genetic composition observed within each population [81,82].

In addition, the significant isolation-by-distance (IBD) pattern suggests that genetic connectivity decreases with increasing geographic distance, which may further emphasize the importance of spatially explicit, population-level monitoring strategies. Long-term ecological monitoring programs may utilize population-level genetic baseline data to evaluate population dynamics, habitat changes, and restoration outcomes [83]. In particular, the heterogeneous patterns of clonal and genetic diversity among populations indicate that genetic composition may not be uniform, even among hydrologically connected habitats [84]. Therefore, future conservation and utilization strategies should consider the historical background and specific habitat context of regional populations.

Overall, the genetic information generated in this study may serve as baseline data for freshwater ecosystem management and for the sustainable utilization of plant resources derived from this species and related taxa.

## 5. Conclusions

In this study, we developed 21 novel genome-derived SSR markers and applied them to evaluate genetic variation, differentiation, and spatial structure of *E. ussuriensis* across 6 populations in South Korea. The markers exhibited sufficient polymorphism and reproducibility, providing reliable tools for assessing population-level genetic patterns in this species.

Genetic and clonal diversity varied among populations, suggesting that habitat context and local population history influence genetic composition. Genetic differentiation was observed among populations, accompanied by a significant isolation-by-distance (IBD) pattern. Despite the lack of direct hydrological connectivity among sites, gene flow appears to be associated with geographic distance and site-specific demographic processes. These patterns are reflected in the spatial genetic structure, suggesting a tendency for populations to form distinct groups. These findings suggest that regional populations of *E. ussuriensis* exhibit heterogeneous genetic characteristics.

Overall, this study provides preliminary insights into the genetic variation in *E. ussuriensis*, contributing to a better understanding of its ecological dynamics. The SSR markers developed here may facilitate future comparative studies and help inform evidence-based conservation and resource management strategies.

## Figures and Tables

**Table 1:** Assessment of genetic variations of 21 microsatellite loci developed from *Eleocharis ussuriensis*.

GenBank Accession No.	Locus	PIC	A/L	Ho	He	F_IS_	F_ST_	HWE
PX920838	EU01	0.861	16	0.492	0.876	0.147	0.342	NS
PX920839	EU04	0.879	16	0.500	0.893	0.152	0.339	ND
PX920840	EU05	0.843	18	0.117	0.862	0.683	0.572	***
PX920841	EU08	0.772	9	0.308	0.801	−0.321	0.708	***
PX920842	EU21	0.802	9	0.600	0.825	−0.806	0.596	***
PX920843	EU23	0.726	8	0.175	0.761	−0.177	0.804	***
PX920844	EU26	0.825	13	0.133	0.848	0.345	0.759	***
PX920845	EU30	0.810	10	0.642	0.831	−0.687	0.541	NS
PX920846	EU31	0.748	10	0.625	0.786	−0.703	0.531	***
PX920847	EU32	0.840	10	0.350	0.859	−0.101	0.629	***
PX920848	EU45	0.744	8	0.517	0.780	−0.519	0.563	***
PX920849	EU48	0.682	9	0.292	0.715	−0.247	0.672	***
PX920850	EU51	0.848	10	0.608	0.865	−0.685	0.581	***
PX920851	EU57	0.465	4	0.267	0.551	−0.259	0.614	***
PX920852	EU58	0.654	5	0.167	0.709	−0.195	0.803	***
PX920853	EU60	0.770	6	0.167	0.804	−0.011	0.794	***
PX920854	EU80	0.794	9	0.125	0.820	0.275	0.789	***
PX920855	EU82	0.882	14	0.525	0.895	−0.480	0.602	ND
PX920856	EU86	0.641	5	0.117	0.682	−0.520	0.887	***
PX920857	EU96	0.594	7	0.200	0.634	−0.198	0.736	***
PX920858	EU100	0.758	5	0.667	0.795	−0.935	0.565	***
	Mean	0.759	9.6	0.362	0.790	−0.250	0.639	

ND, not determined; PIC, polymorphism information content; A/L, number of alleles per locus; Ho, observed heterozygosity; He, expected heterozygosity; F_IS_ and F_ST_, Wright’s F-statistics; HWE, Hardy–Weinberg equilibrium; NS, not significant; *** *p* < 0.001.

**Table 2:** Genetic diversity in 6 populations of *E. ussuriensis* analyzed using 21 microsatellite loci.

Population	N	P (%)	A	Ae/L	A_U_	Ho	He	G/N
YC	20	76.2	59	1.8	28	0.331	0.316	0.95
HC	20	71.4	39	1.7	16	0.645	0.349	0.40
GS	20	33.3	33	1.4	26	0.155	0.163	0.40
JC	20	57.1	41	1.4	16	0.260	0.222	0.60
US	20	47.6	34	1.5	17	0.274	0.224	0.30
BS	20	76.2	70	2.2	45	0.505	0.432	1.00
Mean	20	61.6	46	1.7	24.7	0.362	0.284	0.80
Total	120	100	201	5.4	148	0.362	0.787	0.61

N: Number of samples; P (_%_): Polymorphic loci determined based on the 0.95 criterion; A: Observed number of alleles; Ae/L: Effective number of alleles per locus; Ho: Observed heterozygosity; He: Expected heterozygosity; G: Number of multilocus genotypes; A_U_: Number of unique alleles.

**Table 3:** Results of the Wilcoxon signed-rank test for bottleneck detection under three mutation models, using one-tailed analyses (Hep).

Population	He	IAM	SMM	TPM	F_IS_
YC	0.316	0.355	0.425	0.392	−0.023
HC	0.349	0.257 ***	0.307 ***	0.283 ***	−0.840
GS	0.163	0.310 **	0.372 *	0.343 **	0.076
JC	0.222	0.310	0.383	0.346	−0.142
US	0.224	0.277 **	0.342 **	0.312 **	−0.198
BS	0.432	0.447 **	0.528 *	0.486	−0.145

IAM, infinite alleles model; SMM, stepwise mutation model; TPM, two-phase model; F_IS_, Inbreeding coefficient. *p*-values of expected heterozygosity were obtained from the three models. Significance levels: * *p* < 0.05, ** *p* < 0.01, *** *p* < 0.001).

## Data Availability

The original contributions presented in this study are included in the article. Further inquiries can be directed to the corresponding author.

## Supplementary Materials

The following supporting information can be downloaded at https://www.mdpi.com/article/10.3390/genes17050513/s1. Figure S1. Representative images of *E. ussuriensis* from two of the six sampled populations: (A) habitat view of the YC (Yeoncheon) population, and (B) specimen image from the GS (Goseong) population. The color checker shows millimeters (mm), and the vertical ruler shows centimeters (cm). Table S1. Summary of population information for six *E. ussuriensis* sampling sites in South Korea, representing distinct and hydrologically independent drainage systems. Table S2. Characteristics and primer information for 21 microsatellite loci developed in *E. ussuriensis*. Fluorescent label codes: F = FAM, V = VIC, N = NED, and P = PET. Table S3. Pairwise directional migration estimates among six populations of *E. ussuriensis* inferred using the divMigrate approach. Lower triangle: gene flow; upper triangle (red): geographic distance (km).

## Abbreviations

The following abbreviations are used in this manuscript:

SSR	Simple Sequence Repeat
He	Expected Heterozygosity
Ho	Observed Heterozygosity
PCR	Polymerase Chain Reaction
PIC	Polymorphism Information Content
IAM	Infinite Alleles Model
SMM	Stepwise Mutation Model
TPM	Two-Phase Model
HWE	Hardy–Weinberg Equilibrium
A/L	Number of Alleles per Locus
CI	Confidence Interval
PCoA	Principal Coordinate Analysis
