# Supplementary material for: Genetic Variation and Spatial Genetic Structure of Eleocharis ussuriensis Zinserl. in South Korea: Implications for Ecological Monitoring and Resource Management
Source: Genes (Basel). 2026 Apr 26;17(5):513. doi: 10.3390/genes17050513 (PMC13205643; doi:10.3390/genes17050513)
Supplement: Supplementary file 1 [file genes-17-00513-s001.zip › genes-4240607-supplementary.pdf]

Supplementary

(A)

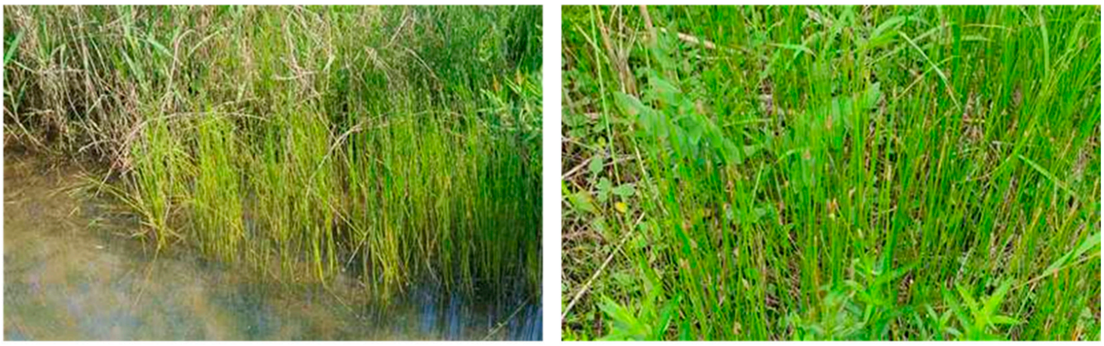

(B)

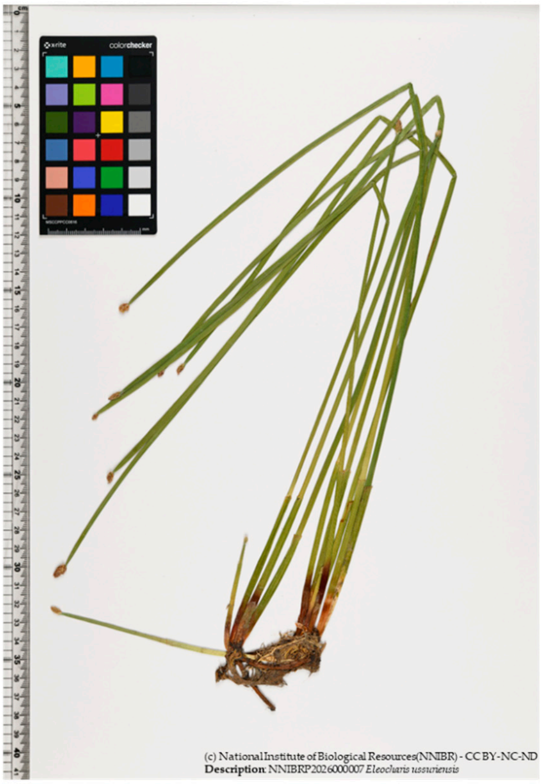

Figure S1. Representative images of *E. ussuriensis* from two of the six sampled populations: (A) habitat view of the YC (Yeoncheon) population and (B) specimen image from the GS (Goseong) population. The color checker shows millimeters (mm), and the vertical ruler shows centimeters (cm).

Table S1. Summary of population information for six *E. ussuriensis* sampling sites in South Korea, representing distinct and hydrologically independent drainage systems.

Population abbreviation	Basin	Habitat type	Hydrological conditions	Sampling area (m)	Sample Size
YC (Yeoncheon)	Imjin River	Riverine (lotic)	Riverine, agriculture, seasonal flooding	350× 120	20
HC (Hwacheon)	Han River	Riverine (lotic)		240 × 50	20
GS (Goseong),	East coastal	Riverine (lotic)	Coastal river, estuarine influence	320 × 150	20
JC (Jecheon)	Drainage system -	Reservoir (lentic)	long-established, managed , anthropogenic	92 × 30<br>90 × 15	20
US (Uiseong)	Nakdong River	Riverine (lotic)	Riverine, agriculture, seasonal flooding	450 ×120	20
BS (Busan)	Suyeong River	Reservoir (lentic)	Protected reservoir, water supply	270 × 70	20

Table S2. Characteristics and primer information for 21 microsatellite loci developed in *E. ussuriensis*. Fluorescent label codes: F = FAM, V = VIC, N = NED, P = PET

Locus	Primer sequence (5′-3′)	Motif	Allele range (bp)	Label
EU01	F: GGCAGGTCTAAGACATCAAGGA<br>R: GCACCAATTGGCAGAGAGAC	(GT)13	346-410	F
EU04	F: CAAGGCCACCTAGCTTGGAT<br>R: CCGTCGCTCCGTTTATTTCG	(GA)13	216-304	N
EU05	F: GCCTTCACCCACTGTAGGTA<br>R: AGCCGCATAGTTCGACACAT	(AT)13	208-298	N
EU08	F: AGGTGATACACGGTGGGAGA<br>R: GTATCGGCGGGTAGTGGAAG	(TC)14	354-402	F
EU21	F: CCCCAGTCCAGGTCAGAAAC<br>R: GTGCATGGGACTGTATCTGAGA	(AG)16	350-388	F
EU23	F: TAGGTCGAGTCCAGCACTGA<br>R: CCTATCCAGTGCACCAAGCT	(GA)16	320-346	F
EU26	F: TGCTCGCATGATTAGCACAC<br>R: AAGGCGTGGAACTGTTGAAG	(TA)17	264-314	N
EU30	F: TTGTGTTTGCGTGCATGTGT<br>R: CTGATGTTGCTTGTCAGCATGTA	(AG)18	247-300	V
EU31	F: AAGAGAGGGGTGCCACATTG<br>R: GCACGTAGGAGTTGAGATGC	(GA)18	266-298	N
EU32	F: CCGACAGGGACCTTCGAAAT<br>R: AACTCCGTATTAGCGACGCA	(AT)19	238-356	F
EU45	F: GGGCCGCATATTGATGGAGA<br>R: CGCAATCATCCCACGGTCTA	(TC)25	276-302	N
EU48	F: CTACGACGTCTGCTGCACTTG<br>R: CATACCAGTACTGGACCTGCATT	(TC)27	248-308	N
EU51	F: ATCTACCCACCATGCTCGTG<br>R: GTGTGTGTGGCAGCAAATCA	(TAT)13	323-371	F
EU57	F: GCCGGAAACACCATGGTCAA<br>R: CGAGTCCCGGCGATCATAAA	(AGA)16	236-242	V
EU58	F: CCCAATCCTCTTTTATAGGCATAC<br>R: CAGGCTACAAATGATGAGCACA	(GAA)17	163-223	P
EU60	F: TGCAACCGGGTCTCAGTTCC<br>R: GCATGTCCAGAGGCTAGTCC	(ATT)18	238-285	N
EU80	F: ACCTGTGACCACCTAGGACA<br>R: GGATGAGCAGATCGAGCCAA	(TCT)35	305-419	F
EU82	F: GGTCTCCTACCTCCTCCGAA<br>R: GGGAAAGGCCTGGGAGAAAA	(CTTT)21	182-254	V
EU86	F: ATGGCTGCTTCGGTCTTTAG<br>R: CCAAACGGAGGAGATTCCAGT	(ATAC)15	178-222	V
EU96	F: GGTAGCGACAAAAGTCAAATTGC<br>R: CTACGGTTGCTTGTGGGTCT	(TCTTT)8	210-241	V
EU100	F: CTGTGCCCAGCAACTTTGTC<br>R: GCGACGCCAATATCACCTTG	(CTTTT)7	250-275	V

Table S3. Pairwise directional migration estimates among six populations of *E. ussuriensis* inferred using the divMigrate approach. Lower triangle: gene flow; upper triangle (red): geographic distance (km).

Population	YC	HC	GS	JC	US	BS
YC	-	57.6	129.8	147.1	240.5	369.1
HC	1.00	-	77.8	109.1	209.0	336.7
GS	0.03	0.03	-	136.9	230.7	351.6
JC	0.82	0.61	0.04	-	100.9	229.3
US	0.15	0.08	0.02	0.08	-	127.5
BS	0.13	0.13	0.07	0.04	0.39	-
